# Advances in the role of ion channels in leukemia

**DOI:** 10.1016/j.heliyon.2024.e33452

**Published:** 2024-06-22

**Authors:** Tianjie Zhu, Jingyuan Zhao, Jinnan Liu, Siyu Tian, Shuai Li, Hong Yuan

**Affiliations:** a Central Hospital of Dalian University of Technology, Dalian, China; b Department of Pharmacy, The First Affiliated Hospital of Dalian Medical University, Dalian, China

**Keywords:** Ion channel, Leukemia, Cell function, Therapy

## Abstract

Ion channels are widely present in cell membranes, serving as crucial pathways for the movement of ions enter and exit cells. Variations in the expression of ion channels are crucial for regulating cellular functions. Among the genes associated with leukemia, certain genes encode ion channels. When these ion channels experience dysfunction or changes in expression, they can impact the physiological functions and signal transduction of hematopoietic cells, thereby regulating leukemia cell proliferation, differentiation, invasion/migration, and apoptosis. This article will provide a comprehensive review of the research progress on the expression and function of various ion channels in leukemia, thoroughly exploring their roles and mechanisms in the onset and progression of the disease, providing new insights and ideas for identifying potential biomarkers and developing new treatment methods for leukemia, thereby promoting innovations in future leukemia diagnosis and therapy.

## 1 Introduction

Leukemia is a malignant disorder of the hematopoietic system in which genetic changes in blood cell precursors lead to tumor changes and clonal proliferation. It is characterized by the progressive abnormal proliferation of one or more undifferentiated cells, extensive infiltration of other organs such as the liver and spleen, and the unchecked proliferation of leukemia cells, which hinders the growth of normal blood cells [1]. In clinical practice, leukemia is primarily classified into four types based on the origin of the leukemia cells and the rate of disease progression: acute myeloid leukemia (AML), chronic myeloid leukemia (CML), acute lymphoblastic leukemia (ALL) and chronic lymphocytic leukemia (CLL). The disease can occur at any age, ranging from newborns to the elderly, but different types of leukemia tend to have different age distributions. For example, ALL is most common in early childhood and is the most prevalent pediatric malignancy. Fortunately, the five-year survival rate has increased from below 50 % in the 1970s to 85 % in the 21st century [2]. CLL almost exclusively occurs in individuals over the age of 40, with a median age at diagnosis of 70 years and this disease primarily affects the elderly population, reflecting a strong correlation between its incidence and age [3,4]. A prognostic model evaluation reported that the 4-year survival rate with the first-line agent acarabitinib was about 88 %, the 2-year survival rate with zarutinib was about 94 %, and the 7-year survival rate after treatment with irutinib was approximately 78 % [5]. AML predominantly occurs in adults, accounting for up to 33 % of cases [6]. It is estimated that in 2024, there will be more than 20,000 new cases in the US, and over 10,000 people will die from the disease. The 5-year survival percentage from 2014 to 2020 was only 31.9 % [7]. According to a study in the United States, 1 to 2 people in every 100,000 adults suffer from CML, accounting for 15 % of new adult leukemia cases. From 2000 to 2022, the estimated number of patients increased from 30,000 to 150,000, an increase of about 8600 cases per year. The 10-year survival rate for CML has increased significantly, rising from around 20 % to between 89 % and 90 % [8]. Part of the causes of leukemia are related to previous chemotherapy, environmental radiation and exposure to chemical reagents, but the main cause is chromosomal variation or a certain gene mutation that leads to genetic changes [9]. Indeed, the exact pathogenesis behind leukemia remain unclear. Therefore, understanding the causes of leukemia, identifying biomarkers for early diagnosis, and finding new pharmacological targets are particularly important.

**Fig. 1:**
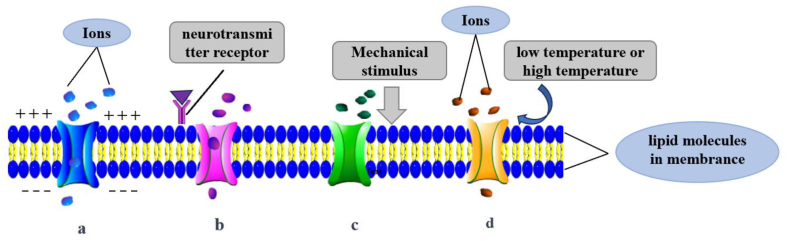
Ion channels are classified by gated regulation a. Voltage-Gated Ion Channels open and close in response to changes in the cell membrane's electric potential. They are regulated by variations in membrane voltage. **b.** Ligand-Gated Ion Channels open and close upon binding of specific chemical substances (ligands), such as neurotransmitters. An example is the acetylcholine receptor, which responds to acetylcholine. **c.** Mechanosensitive Ion Channels are regulated by mechanical forces, responding to physical pressure or tension. **d.** Temperature-Gated Ion Channels change their states in response to temperature variations.

Ion channels, with their selective permeability, are essentially membrane proteins that can be readily detected in leukemia cells. The expression of ion channels can regulate the proliferation, invasion or apoptosis of leukemia cells, thereby affecting the progression of the disease [10]. Ion channels play a pivotal role in processes such as neurotransmission, muscle contraction, and cell proliferation, and are crucial for maintaining normal cellular physiological functions. Their significance cannot be overstated, as they are essential for the proper functioning of cells and, consequently, the overall health of the organism [11]. For example, TRPV2 has a variety of functions as a molecular sensor, including phagocytosis, migration/chemotaxis, cytokine secretion, tissue infiltration. Circulating lymphocytes are affected by fluid flow, osmotic pressure, blood pressure changes, and shape alterations during processes such as tissue extravasation, antigen recognition, maturation. TRPV2 can directly or indirectly regulate these processes through mechanical stretching [12]. The deletion, acquisition, or mutation of the TRPV2 gene can affect the growth, differentiation, and survival of tumor cells, thereby promoting the formation and progression of hematological tumors, and playing a crucial role in their development. Ion channels are classified in various ways, with one primary method according to their gating mechanism（Fig. 1）.Voltage-Gated Ion Channels: These channels are a type of ion channel sensitive to changes in the cell membrane potential which open or close in response to changes in membrane potential, allowing specific ions to pass through the cell membrane, thereby regulating the cell's electrical activity. Examples include sodium channels and potassium channels, primarily involved in neurotransmission and muscle contraction. Ligand-Gated Ion Channels: These channels open and close in response to the binding of specific chemical substances (ligands). When a ligand binds to the receptor site on the channel, it causes a conformational change in the channel, allowing it to open or close and permit specific ions to pass through the cell membrane. For example, neurotransmitter receptor channels, such as the nicotinic acetylcholine receptor, and various other neurotransmitter channels belong to this category. Mechanosensitive Ion Channels: These channels are regulated by mechanical forces, responding to physical pressure or tension, and are essential in cells that detect mechanical stimuli, such as those involved in hearing and touch. Temperature-Gated Ion Channels: These channels are highly sensitive to temperature changes. Members of the TRP ion channel superfamily, such as TRPV1 and TRPM8, open in response to high or low temperatures. Another important classification method is based on the type of ions they transport K^+^, Cl^−^, Ca^2+^ and Na ^+^ are the most critical ions involved in cellular activities, significantly impacting cellular physiological functions. Studies have proven that the expression and functional changes of almost all types of ion channels, including these four ion channels, can affect tumor progression, metastasis, and drug resistance [13].

In human breast cancer, TRPM7 is a protein with high permeability to divalent cations such as Ca^2+^ and Mg^2+^. The activity of the TRPM7 channel is involved in cell proliferation and tumor growth by regulating calcium concentration, while its calcium flux plays a role in modulating cellular resistance to external pressure and epithelial-mesenchymal transition (EMT). Additionally, the intrinsic serine/threonine kinase activity of TRPM7 is crucial for EMT, cell migration, and the development of tumor metastasis [14]. TMEM16A, also known as ANO1, is a transmembrane protein belonging to the TMEM16 family. TMEM16 primarily functions as a calcium-dependent chloride channel, which opens in response to increased intracellular calcium levels, allowing chloride ions to pass through the cell membrane. Located in the amplification region of chromosome 11q13, it contains various proteins associated with the cancer cell cycle, proliferation, and apoptosis, including cyclin D1, FGF19, FGF4, and Fas-associated death domain. TMEM16A is involved in the growth of various cancer cells, including esophageal and bladder cancer [15]. Kca and Kv1.1 channels have also been shown to control the proliferation of colon cancer and prostate cancer tumor cells by modulating membrane potential [16,17]. In prostate cancer and breast cancer, voltage-gated sodium channels (VGSC) increase the invasiveness of tumor cells by stimulating cysteine cathepsin activity [18]. At present, research on the role of ion channels has gradually shifted from excitatory cells such as neurons and hearts to solid tumor cells. Leukemia-initiation cells (LICs) are a small group of cells involved in the initiation, development and resistance of AML. Ligand ion channels (P2Xs) are cation-selective channels permeable to K^+^, Ca^2+^, and Na^+^, which are widely expressed in hematological diseases and solid tumors, with ATP being the only known ligand [19]. It has been reported that P2X1 can enhance the self-renewal of LICs through PBX3-BCAT1 pathway and promote the occurrence of leukemia, and the P2X1 antagonist PPNDS can significantly reduce the proliferation of AML cells, suggesting that P2X1 may be an effective way to treat AML [20]. This review analyzes the expression of various ion channels in different diseases, with a focus on leukemia, and examines their roles and mechanisms in the proliferation, differentiation, invasion/migration, and apoptosis of different types of leukemia cells. By summarizing and discussing existing data, the conclusion is drawn that ion channels may serve as novel biomarkers for leukemia diagnosis and provide reasonableinsights for identifying new therapeutic targets.

## 2 The role of different types of ion channels in leukemia

### 2.1 Potassium channel

**Fig. 2:**
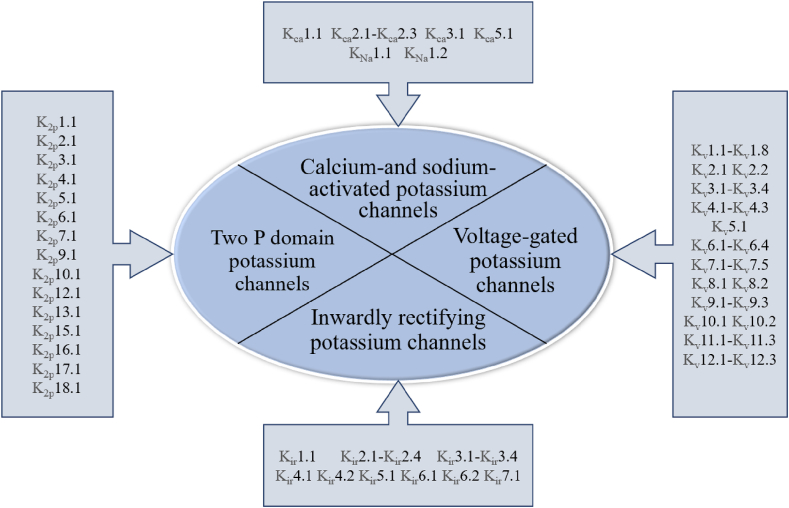
Main types of potassium ion channels Potassium channels are currently classified into four categories: voltage-gated (K_V_), calcium - and sodium-activated (K_ca_/K_Na_), inward rectified (K_ir_), and two-pore domain (K_2p_). Among them, the voltage-gated potassium channel is subdivided into 12 types K_V_1-K_V_12, the calcium and sodium activated channel is subdivided into 6 types, the inward rectifier channel is subdivided into 7 types, and the double-pore domain channel is subdivided into 18 types.

Potassium ion channels are very common and diverse type of ion channels. They have numerous important functions within living organisms, including regulating cell membrane potential, controlling action potentials in nerves and the heart, and participating in the release of hormones and neurotransmitters. The International Union of Basic and Clinical Pharmacology (IUPHAR) has proposed a standardized nomenclature system for potassium channels which classifies cloned channels into different categories based on their gene families and structural characteristics. According to the number of transmembrane domains, potassium channels are categorized into 2TM (2 transmembrane domains), 4TM (4 transmembrane domains), and 6TM (6 transmembrane domains). Potassium channels have been classified into four categories （Fig. 2）: voltage-gated (K_V_), calcium and sodium-activated (K_ca_/K_Na_), inwardly rectified (K_ir_), and biporous domain (K_2p_) [21].

At present, hEAG1 (K_V_10.1) is one of the most studied channels, and it has been proved that it is overexpressed in various solid tumors, including liver cancer [22], cervical cancer [23], breast cancer [24] and colorectal cancer [25]. These studies indicate that hEAG1 channel can function as potential tumor marker [26]. In addition to expression in solid tumors, hEAG1 has also been found to be significantly upregulated in hematologic cancers such as AML, CML, and MDS. In addition, it has been observed that a close correlation between the expression of hEAG1 channels and increasing patient age. This elevated expression level is not only associated with a higher relapse rate but also leads to a significantly shortened overall survival time [27]. hEAG1 channel activity can be regulated by the tyrosine residues Tyr90, Tyr344, and Tyr485 of the EGFR kinases, which may further modulate tumor growth [28]. Additionally, studies have demonstrated that the p53/miR 34/E2F pathway regulates the expression of hEAG1. This regulatory network plays a critical role in controlling cell cycle progression and apoptosis, linking hEAG1 expression to cancer development and progression [29]. In early reports, it was found that myeloblastic leukemia cells ML-1 can express functional potassium ion channels that are sensitive to 4-AP when they proliferate. Treatment with 4-AP can cause ML-1 cells to arrest at the G1 phase of the cell cycle [30,31], so it can be inferred that potassium ion channels in ML-1 cells are closely related to the cell cycle. The hERG1 (K_V_11.1) potassium channel, part of the EAG family, is abundantly expressed in leukemia cells. It influences cell proliferation, apoptosis, migration, and angiogenesis, playing a crucial role in promoting cancer progression. Pillozzi et al. [32] discovered that specifically blocking hERG1 channels causes AML cells to arrest in the G1 phase and reduces their migration on a fibronectin-containing extracellular matrix. This indicates that hERG may simultaneously affect both the cell cycle and migration. This effect is triggered by cell adhesion and is dependent on a signaling pathway centered around the serine/threonine kinase AKT, regulated by hERG1 channel activity. Thus, it is evident that these two related channels play similar biological roles in leukemia.

K_V_1.3 is another member of the voltage-gated potassium channel family, first discovered on the plasma membrane of human T lymphocytes. This channel plays a critical role in the regulation of immune cell function, particularly in T cell activation and proliferation [33]. Extensive research has found that K_V_1.3 channels are expressed in many tissues, including human B lymphocytes, macrophages and the brain. They are present in the inner mitochondrial membrane of leukemia T lymphocytes, prostate cancer cells, and breast cancer cells [34]. These channels are closely associated with cell proliferation and apoptosis. Other studies have indicated that K_V_1.3 can directly interact with Bax in lymphocytes. This interaction is believed to play a critical role in regulating apoptosis, linking the function of K_V_1.3 channels to the apoptotic pathways in these cells. K_V_1.3 inhibitors cause hyperpolarization of the inner mitochondrial membrane through Bax/Bac, releasing reactive oxygen species (ROS) and cytochrome *c*, which in turn leads to cell death [35,36]. For another, Fas receptors can regulate the function of channels such as K_V_1.3 and other channels on the cell membranes influencing cell apoptosis by interacting with potassium outflow, cell contraction, and the caspase family [37]. In terms of cell proliferation, research indicates that K_V_1.3 and calcium-activated potassium channels are poorly expressed in resting CLL cells, but highly expressed in activated cells, indicating that K_V_1.3 is involved in inducing the proliferation of CLL cells. This differential expression underscores the importance of Kv1.3 in the activation and growth of leukemia cells, highlighting its potential as a therapeutic target for controlling CLL cell proliferation [38,39]. The two-pore domain potassium channels K_2P_ regulate cell proliferation, differentiation, and apoptosis by changing certain signaling pathways in cells. Among them, K_2P_18.1 (TRESK) is associated with immune dysfunction and is involved in the proliferation of ALL cells as well as the expression of various genes [40]. It is considered a potential target for cancer therapy.

### 2.2 Calcium channel

Intracellular calcium ions are one of the most crucial second messengers, playing a crucial role in cell proliferation, differentiation, migration, and apoptosis. Calcium channels include the TRP superfamily of transient receptor potential channels (TRPC, TRPM, TRPV, TRPA, TRPP, TRPML) and voltage-gated calcium channels (Ca_V_1–Ca_V_3), continuous calcium influx is an important mechanism for regulating the production, proliferation, and differentiation of cytokines in blood cells [41].

TRPM2 is a Ca^2+^ -permeable cation channel sensitive to heat and ROS that plays a vital role in cell survival and in the adaptive response of various cell populations during oxidative stress. TRPM2 is highly expressed in several types of cancer, including breast, pancreatic, leukemia, suggesting that it enhances the survical capacity of cancer cells [42]. TRPM2 can maintain cancer cell viability by preserving mitochondrial function, producing ATP, reducing cellular ROS levels, and facilitating DNA repair [43]. When TRPM2 is inhibited, it disrupts the G2/M cell cycle arrest, leading to cell death. Additionally, this inhibition enhances the sensitivity of several malignancies, including T-cell leukemia, to chemotherapeutic agents. This dual role of TRPM2 in cell viability and drug sensitivity highlights its potential as a therapeutic target in cancer treatment [44,45]. In addition, genetic polymorphisms of TRPM5 have been found to be closely associated with the risk of developing childhood leukemia, and this polymorphism may increase the likelihood of developing leukemia by affecting the function of ion channels, thereby altering intracellular calcium signaling and other crucial biological processes [46]. TRPM7 is localized on the cell membrane of human chronic myeloid leukemia cell K562, which is a spontaneously activated Ca^2+^ internal circulation pathway and is important for maintaining Ca^2+^ homeostasis. Both activation and inhibition of ERK are known to promote differentiation of stem/progenitor cells into mature erythrocytes. It should be emphasized that the TRPM7 channel is essential for maintaining ERK activity to promote K562 cell proliferation, both before and after heme stimulation [47].

Calcium channel TRPV1 is a ligand-gated cation channel that affects proliferation signaling and cell death pathways mainly by changing calcium influx. TRPV1 can inhibit the growth of chronic myeloid cells and promote leukemia cell apoptosis, and its activation can induce calcium influx, oxidative stress, endoplasmic reticulum stress, mitochondrial dysfunction, and caspase activation. Therefore, activating TRPV1 can enhance conventional treatments and improve the management of chronic myeloid leukemia [48]. TRPV5 and TRPV6 are highly expressed in malignant states of human lymphocytes and leukemia Jurkat T cells, where they regulate cell proliferation [49]. Other studies have shown that TRPV5 and TRPV6 are expressed in leukemia cell K562, and the increase in channels activates Ca^2+^/calmodulin-dependent kinase II (CaMKII), thereby increasing intracelluar Ca^2+^, regulating cell proliferation, differentiation, and resistance to apoptosis [50,51]. Further research has revealed that the expression of TRPV5/6 channels associated with cell proliferation and differentiation is regulated by 1,25-dihydroxyvitamin D3 (1,25-(OH)2D3). This compound not only inhibits proliferation but also induces the differentiation of leukemia cells. For example, it induces the differentiation of human bone marrow monocytes U937 into monocytic/macrophage. These findings suggest that TRPV5/6 channels can modulate leukemia cell differentiation in a manner dependent on 1,25-(OH)2D3-dependent [49].

### 2.3 Sodium channel

VGSCs (Voltage-gated sodium channels) are a class of membrane proteins that are widely present in nerve and muscle cells, distributed across the membranes of most excitable cells. These channels play a crucial role in maintaining the cell's electrophysiological properties and in the generation and propagation of action potentials. By regulating the permeability to sodium ions, they are key to the transmission of nerve signals and the regulation of muscle contraction [52]. VGSCs channels are classified into two types, TTX-sensitive (Na_V_1.1–1.4, Na_V_1.6, Na_V_1.7) and TTX-tolerant (Na_V_1.5, Na_V_1.8, Na_V_1.9). Tetrodotoxin (TTX) is a selective blocker of sodium channels, capable of inhibiting the expression and function of sodium channels, thereby reducing the metastatic and invasive abilities of cancer cells. Several studies have demonstrated that VGSCs are expressed in various tumors, including cervical, prostate, and breast cancer. They can regulate tumor cell growth, invasion, and metastasis [[53], [54], [55]]. However, the role of VGSCs in leukemia has been poorly studied. Scott et al. found that there were four channels expressed in JurkatT lymphocytes: Na_V_1.3, Na_V_1.5, Na_V_1.6 and Na_V_1.7, of which Na_V_1.5 was the main subtype. They also found that TTX could significantly inhibit invasion of cells, reaching 93 %. As TTX is a highly specific VGSCs blocker, it indicates that the activity of VGSCs channels can promote the invasion of Jurkat T cells, which is required for T lymphocytes to respond to intravascular congestion and inflammation [56,57]. Interestingly, Roger et al. [58] conducted experiments using Jurkat cells and found that cell invasiveness was reduced by approximately 93 % after TTX treatment. This suggests that the sodium currents observed in leukemia cells may be due to the upregulation of already expressed channels.

The biophysical properties of another class of natural, non-voltage-gated sodium channels (NVGS) in human lymphoma cells U937 shares similar biophysical properties with epithelial sodium channels (ENaCs). The activity of NVGS channels has been shown to be heavily dependent on the organization of the actin cytoskeleton. However, known DEG/ENaC inhibitors, such as amiloride, are unable to block NVGS channels in U937 cells [59]. The expression of VGSCs and NVGS may be related to the invasiveness of solid tumor cells, while their expression under normal conditions might be a mechanism by which lymphocytes invade infected tissues. Therefore, further research is needed to explore the potential application of sodium channel blockers in leukemia.

### 2.4 Chloride channel

While the research on chloride ion channels came relatively late, Cl^−^ channels also play essential biological roles within cells, such as regulating cell size, nerve excitation, and organelle acidification. Mutations in chloride channels can lead to diseases such as cystic fibrosis, myotonia, and kidney stones, and GABA(A) (γ aminobutyric acid A) receptor chloride channel modulators have been used in clinical practice [60]. The biological function of Cl^−^ channels in lymphocytes and neutrophils is not clear. However, studies have found that chlorine channel blockers such as NPPB, 9-AC, and tamoxifen can inhibit the proliferation and cell cycle progression of human leukemia cells. NPPB has the strongest inhibitory effect, which can block leukemia cells in the G0/G1 phase and induce the expression of P21 [61]. Chloride intracellular ion channel CLICs are organelle ion channels, and the CLIC protein family includes CLIC1-CLIC6. Numerous studies have proved that CLIC4 can regulate cell proliferation, differentiation, apoptosis and other functions [62]. p53, c-Myc, and TGF-β can regulate CLIC4, which can induce the NF-κB-dependent activation of hypoxia-inducible factor (HIF) and, through its microenvironmental functions, participate in tumor progression. CLIC4 is significant in the pathogenesis of AML. Huang et al. [63] pointed out that the oncogene miR-181 correlates positively with CLIC4, while the tumor-suppressing gene miR-10a correlates negatively with CLIC4, indicating that CLIC4 could be a novel and potential adverse prognostic factor and therapeutic target for CN-AML (cytogenetically normal AML) warrants further investigation.

CLC anion transporters are widely present in mammals. Among them, CLC-1, CLC-2 and CLC-Ka/-Kb are plasma membrane chlorine channels, while CLC-3 to CLC-7 are 2Cl^-^/H^+^ exchangers on the membranes of endolysosomes. CLC Cl^−^ can regulate electrical excitability, intracellular and extracellular ion homeostasis [64]. We know that the reduction in cell volume is a key factor in inducing apoptosis, and this is caused by the efflux of ions, which is due to the increased conductance of K^+^ and Cl^−^ [65]. Many studies have reported that apoptosis stimulates the activation of Cl^−^ currents [66,67]. Whereas the anion conductance that triggers apoptosis is regulated by volume-sensitive outward rectified Cl^−^ channels. Jiang et al. [68] found that CLC-2 is weakly expressed in immune cells, CLC-4 is mainly expressed in B cells, and CLC-3 is widely expressed in leukemia cell lines, T/B cells, and neutrophils. The distribution characteristics of different chloride channels in different immune cells indicate a unique role and provide a potential therapeutic pathway for leukemia. CLC-3 is the focus of current research. Research has revealed that in K562 cells, oxidative stress induces an increase in CLC-3-dependent anion channels activity and permeability, leading to the expulsion of potassium chloride and water from cells, subsequently causing cell contraction and apoptosis [69]. It indicates that CLC-3 may have an important relationship with leukemia cell contraction and apoptosis.

## 3 The role of ion channels and membrane potentials in cancer

In biology, the potential difference refers to the electric potential difference between the inside and outside of the cell membrane, commonly referred to as the membrane potential. Ion channels are present on the cell membrane, allowing ions to move freely between the inside and outside of the cell, thereby maintaining the membrane potential difference. This potential difference significantly influences the physiological functions of the cell, including the excitability of nerve cells and the contraction of muscle cells. All depolarization or hyperpolarization of the membrane potential is caused by the movement of ions across the membrane, for instance, the internal flow of sodium ions reduces the negative voltage within the membrane, causing depolarization, while the outflow of potassium ions or the internal flow of chloride ions causes hyperpolarization, that is, increasing the negative voltage within the membrane relative to the external liquid. Ions move rapidly in and out of cells through ion channels, which are pores through which ions formed by transmembrane proteins pass.

A report by Alza et al. indicates that when cells are exposed to hypoxic environments or chemotherapeutic drugs, the voltage-gated calcium channel Ca_V_3 subfamily acts as a key regulator of the G1-S phase transition and stress adaptive signaling [70]. The transition from G1 phaseto the S phase requires Ca^2+^ to flow through multiple Ca_V_3 channels in plasma to promote proliferation, and K_V_ channels and KCa channels also increase their expression. This coordinated regulation of ion channels ensures that cells progress through the cell cycle efficiently, facilitating necessary cellular functions such as proliferation and adaptation to stress conditions. In cancer cells, although Ca_V_3 activity is limited to a small subset of channels expressed in the membrane, it triggers a positive feedback loop that induces initial changes in membrane hyperpolarization. In the 1970s, researchers identified a link between voltage and cell proliferation, leading to the development of innovative cancer treatments involving electric and electromagnetic fields. Currently, four methods are used in cancer treatment:electrochemical therapy, irreversible electroporation, gene electrotransfer, and calcium electroporation. These fields are typically generated between multiple electrodes placed near the tumor. By increasing membrane permeability, electromagnetic fields alter the electrical properties of cells, allowing more ions to enter. This results in changes to membrane potential and cytoplasmic pH, which can trigger apoptosis through various pathways [71].

## 4 Ion channels and the treatment of leukemia

Leukemia is a malignant disease characterized by significant heterogeneity in treatment response and survival outcomes. As molecular diagnostic techniques have advanced, microarray technology can now be used to identify unique gene expression profiles associated with prognosis. This allows for more precise prediction of disease progression and personalized treatment strategies [72]. Historically, ion channel research has primarily focused on identifying the expression and function of these channels in various tumor cells. However, recent studies are increasingly targeting specific ion channels as potential biomarkers for leukemia. This shift aims to enhance diagnostic accuracy and improve targeted therapy options for the disease. As an example, the expression of hEAG1 is associated with increasing patient age, higher recurrence rates, and shorter overall survival. In AML, the expression level of hEAG1 is considered an independent predictor of reduced event-free survival and overall survival rates [73]. Furthermore, ample evidence suggests that hERG1 is a potential target for cancer therapy. hERG1 antagonists can inhibit the proliferation of leukemia cells and suppress the secretion of VEGF-A in myeloid leukemia cells in vitro, and they also exhibit similar inhibitory effects in vivo [74]. Imatinib markedly diminishes hERG current in cells, alongside suppressing the growth and apoptosis of CML cells and the release of VEGF, emphasizing the hERG1 K^+^ channel as a treatment target for CML therapy. It has been documented that hERG1 is significantly overexpressed in primary CML cells and K562 cells, and imatinib can downregulate hERG1 by modulating mRNA and protein levels. It is well known that changes in the concentration of PTK inhibitors can regulate the normal activity of various ion channels in cells. Imatinib, currently the preferred PTK inhibitor for treating CML in clinical settings, has shown excellent therapeutic effects by targeting protein tyrosine kinases such as BCR-ABL and PDGFR [75].

The development of targeted drugs for ion channels has opened new avenues for leukemia treatment, as numerous studies indicate that ion channels are among the most promising targets for anti-leukemia therapy. This potential stems from the involvement of various ion channels and ion pumps in the genes associated with leukemia, highlighting their critical role in the disease's progression and making them attractive candidates for therapeutic intervention. Selective blockers are inhibitors specifically designed to target certain ion channels. Leveraging their advantages allows for the development of novel compounds with specific target selectivity, which is typically achieved through methods such as high-throughput screening, chemical modifications and bioengineering techniques. By precisely targeting ion channels, these selective blockers demonstrate great potential in advancing the treatment of leukemia and other diseases [76]. Different drugs can influence ion channels in various ways, such as blocking conduction pores, competing for agonist binding sites, and altering the likelihood of allosteric transitions between different conformational states [77]. For example, in animal models, there is a drug capable of reducing the functional activity of the highly oncogenic K_V_10.1, and it is essentially a specific monoclonal antibody. This drug can inhibit cancer cell proliferation, slow down tumor growth, and exhibits no significant side effects [78]. Animal venom is a rich source of natural peptides, and peptides derived from various animals, including cone snails, scorpions, anemones, and snakes, have been extensively used in toxin-based drug development. Many of these venom-derived peptides have progressed to clinical trial stages, demonstrating their potential as therapeutic agents [79]. In addition, E4031, a hERG1 blocker, can reduce the proliferation of leukemia, gastric and neuroblastoma cells in vitro [80,81]. It also inhibits the invasiveness of colorectal cancer cells [82], as well as has been found to have similar effects on cells in vivo. It can be used as therapeutic targets for certain tumors. This suggests that E4031 has the potential to inhibit the growth of various cancer types by targeting hERG1 channels, highlighting its therapeutic promise. However, channel blockers also come with drawbacks as they can lead to severe side effects when used, and the careful consideration of these potential adverse effects is crucial in their application. In a classic example, inhibition of K_V_11.1 channels by hERG1 blockers can delay cardiomyocyte repolarization, and the excessive lengthening of the QT interval may lead to fatal fibrillating ventricular arrhythmias [83]. Therefore, it is necessary to consider not only how to apply different blocking mechanisms to cancer treatment, but also the strategies to minimize the risk of side effects. The effectiveness of targeting ion channels for leukemia treatment still requires extensive experimental and clinical research to elucidate the specific mechanisms of interaction between leukemia and ion channels. Understanding these mechanisms will help determine the therapeutic potential and clinical application of ion channel inhibitors in leukemia treatment.

## 5 Ion channels and disease prognosis

With the continuous updating of chemotherapeutic drugs and improvement of nursing care, the cure rate of acute leukemia, especially acute lymphoblastic leukemia, which has a high prevalence in children, has increased significantly. However, a major obstacle to curing acute leukemia, significantly impacting its treatment and prognosis, is the development of resistance to chemotherapy drugs [84]. Leukemia is a disease that is markedly heterogeneous with respect to both therapeutic effects and survival, therefore, the outcome and prognosis of acute leukemia are influenced by patient age, cytogenetics, and disease status [85]. It has been proved by animal experiments and early clinical trials that acting on ion channels alone or combining chemotherapeutic drugs to target ion channels at the same time can affect the therapeutic effect of leukemia patients, so it can be assumed that targeting ion channels improves the chemotherapeutic response of leukemia patients. There is currently little literature on the risks, therapeutic response, and prognosis of targeted ion channel drugs for leukemia. Previous studies have demonstrated that ion channel-targeting drugs can be used alone to treat leukemia. Additionally, to overcome chemotherapy resistance, these drugs can also be combined with chemotherapy agents. Developing ion channel-targeting drugs for leukemia treatment still faces challenges, however, the creation of new targeted therapies must meet two essential conditions: (1) a high specificity for leukemia-related ion channels. (2) No substantial toxic side effects on normal cells.

TRPM8 is significantly overexpressed in AML patients, exerting its oncogenic effects by regulating the ERK-CREB/c-Fos signaling pathway. Inhibiting TRPM8 can reverse the overactivation of ERK signal. Tegaserod maleate exhibits strong anti-AML effects by targeting TRPM8, This demonstrates that TRPM8 is a regulator of leukemia occurrence and has the potential to treat AML as a novel prognostic factor [86]. A recent report by Zhou et al. [87] demonstrated that KCND2 is highly expressed in gastric cancer patients and is associated with poor clinical features and prognosis. Additionally, KCND2 is significantly downregulated in patients treated with anti-PD1 and anti-CTLA4 therapies. These evidences suggest that high levels of KCND2 may adversely affect the efficacy of immunosuppressive treatments. These findings could aid in developing personalized treatment strategies and identifying biomarkers to predict patient responsiveness to immunotherapy. Several studies have demonstrated the expression and regulation of CLIC4 in tumors. Of the 21 patients with colon cancer, 67.2 % were CLIC4 positive, and CLIC4 expression in colorectal cancer is thought to be related to the distribution of tumor stem-like cells [88]. Zou et al. [89] indicated that the expression of CLIC4 is significantly elevated in colon cancer. The expression of CLIC4 is significantly correlated with tumor grade, tumor invasion, and poorer overall survival. Therefore, the expression levels of impenetrable ion channels are different in different tumors, and these evidences are very likely to be valuable prognostic indicators in cancer diagnosis.

## 6 Conclusion

In summary, the regulation of cell function by ion channels primarily occurs through the modulation of cell membrane potential. While previous research primarily focused on the field of electrophysiology, more recent studies have highlighted the significant relationship between ion channels and tumor development and progression. Ion channels influence vital cellular activities such as proliferation, cell cycle regulation, and apoptosis, thereby playing an significant role in leukemia. This growing body of research underscores the potential of ion channels as therapeutic targets in cancer treatment (Table 1). The reasons why ion channels are currently the focus of extensive research are mainly as follows: First of all, because they are expressed in almost all cells and are relatively easy to obtain, when ions enter and exit cells through ion channels, membrane potential can be generated, and electrical signal cells participate in the development of cells, which is the most basic reason. Secondly, with the development of ion channel blockers, many studies have found that the application of ion channel blockers can treat tumors. By changing ion channel activity, blockers affect the distribution of ions inside and outside cells and the physiological function of cells, thereby reducing tumor cell proliferation or promoting apoptosis and other behaviors. Therefore, ion channels have the potential to be used as biomarkers, but due to the complexity of ion regulation mechanisms and the diversity of cell types, the specific mechanisms still need to be further studied.

**Table 1:** Expression and function of ion channels in leukemia.

Ion channel	Disease	Expression	Function	References
K^2+^ channels				
K_V_10.1 (hEAG1)	AML,CML,MDS	↑	Induce cell proliferation, Increase recurrence rate and shorter overall survival	[[27], [28], [29]]
K_V_11.1 (hERG1)	AML,B-CLL,B-ALL,CML	↑	Induce cell proliferation, apoptosis, migration and angiogenesis	[32]
K_V_1.3	CLL,AML,ALL	↑	Induce cell proliferation and Influence apoptosis	[[33], [34], [35], [36], [37], [38], [39]]
K_2P_18.1	ALL	↑	Induce cell proliferation	[40]
Ca^2+^ channels				
TRPM2	T cell leukemia cells	↑	Promote cell apoptosis	[44,45]
TRPM5	ALL	↑	Induce cell proliferation	[46]
TRPM7	CML	↑	Induce cell proliferation	[47]
TRPM8	AML	↑	Induce cell proliferation	[86]
TRPV1	ALL,CML		Induce cell apoptosis and anti-proliferation	[48]
TRPV5	K562 and Jurkat T cell lines	↑	Increase LCs proliferation and anti-apoptosis	[[49], [50], [51]]
TRPV6	K562 and Jurkat T cell lines	↑	Increase LCs proliferation and anti-apoptosis	[[49], [50], [51]]
Na^+^ channels				
Na_V_1.3	Jurkat T cell lines	↑		[56,57]
Na_V_1.5	Jurkat T cell lines	↑	Promote cell invasion	[56,57]
Na_V_1.6	Jurkat T cell lines	↑		[56,57]
Na_V_1.7	Jurkat T cell lines	↑		[56,57]
ENaCs	U937 cell		Associated with cell invasion	[59]
Cl^−^ channels				
CLIC4	AML	↑	Associated with poor prognosis	[63]
CLC-3	CML	↑	Be associated with apoptosis of LCs	[64,68,69]
CLC-4	B cell leukemia cells	↑	Be associated with apoptosis of LCs	[64,68]

Despite numerous studies exploring ion channels as potential markers and therapeutic targets for cancer, most are confined to basic research on their structure, function, pathophysiology, and drug development, with little analysis of patient samples. Violeta [90] investigated the differential expression of ion channels and transporters in the development of hepatocellular carcinoma using rat and human cancer tissues. They discovered that Kcnn4 and Scn2a1 channels exhibited similar expression levels in both non-tumor and tumor tissues, while the Kcna3 potassium channel were significantly overexpressed in tumor tissues. These findings suggest that the genes mentioned above are potential early biomarkers or therapeutic targets for hepatocellular carcinoma.

Ion channels, except as directly facilitating ion transport, can also indirectly affect the sensitivity of leukemia cells to chemotherapy drugs by influencing resting membrane potential and ion gradient changes. Some drugs have been studied for their role in modulating ion channels to treat leukemia. For example, the interaction between scorpion venom toxin BmKKx2 and the hERG potassium channel can induce apoptosis in K562 cells [91]. Recombinant mambalgin-2 can be considered a prototype for a novel medicine for the treatment of CML [92]. The K_V_1.3 channel in acute leukemia cells can be inhibited by Mexiletine, and when used in combination with cytarabine, it further promotes the death of acute lymphoblastic and myeloid leukemia cells [93]. These drugs regulate the growth and differentiation of leukemia cells by affecting ion concentrations or modulating ion channels. Effective drug compounds can be selectively used for targeted therapy of the disease but it is essential to establish the efficacy and safety of these treatments to enhance therapeutic outcomes and minimize adverse effects. This includes extensive clinical trials to evaluate the long-term benefits and potential risks, as well as studies to understand the underlying mechanisms and to identify any possible side effects or drug interactions.

In summary, research on ion channels is of great significance for understanding the mechanisms of life activities, the development of diseases, and drug development. Research on ion channels in leukemia opens new avenues for identifying potential biomarkers, evaluating their pharmacological effects, and developing improved and selective treatment strategies. This represents a significant direction for future research, emphasizing the importance of ion channels in both basic and applied biomedical sciences.

## Funding

This work was supported by grants from the Foundation of 10.13039/501100007620Department of Education of Liaoning Province (LJKZ0862).

## Data availability statement

Data sharing is not applicable to this article as no new data were created or analyzed in this study.

## CRediT authorship contribution statement

**Tianjie Zhu:** Writing – original draft. **Jingyuan Zhao:** Writing – review & editing. **Jinnan Liu:** Methodology, Investigation. **Siyu Tian:** Visualization. **Shuai Li:** Project administration, Funding acquisition. **Hong Yuan:** Supervision, Resources, Conceptualization.

## Declaration of competing interest

The authors declare that they have no known competing financial interests or personal relationships that could have appeared to influence the work reported in this paper.
